# Developing qualitative methods - or “same old wine in a new bottle”

**DOI:** 10.3402/qhw.v10.27679

**Published:** 2015-05-13

**Authors:** Carina Berterö

**Affiliations:** Linköping University, Linköping, Sweden

An ongoing process in qualitative research aims at contributing to a deeper understanding of human experiences and behaviour, and the behavioural world of applied practice. This process also aims to improve the usefulness of the findings by giving evidence for practical action. Interpretive description, an alternative to the conventional qualitative approaches, can help in this regard by providing a better understanding of complex experiential clinical/practical phenomena (Thorne, 2008).

Modifying/expanding a qualitative method, often by adding something, is rather a common phenomenon (see Berterö, 2012, in this journal) when giving examples of grounded theory; another example is phenomenology. Phenomenology is often presented as descriptive or interpretive, but when combining these perspectives or adding something to it, there is not a new method but phenomenology as a movement (Rapporta & Wainwright, 2006). Qualitative content analysis is also a method that has been used as a movement. As Hsieh and Shannon (2005) write, content analysis can be directed, conventional or summative, or as other researchers have stated, deductive or inductive (Elo & Kungäs, 2007), or manifest or latent (Graneheim & Lundman, 2004). These examples of methods that have been modified or expanded have ontological and epistemological standpoints embedded in the different directions of their method and need to be reflected upon by those who conduct the research. So, have the methods mentioned above been expanded and modified, but not developed? I would say that they have been developed; they are in a process of growth.

Sally Thorne (2008) argued that over the past two decades, researchers in the health area have challenged the dominant paradigm of positivistic research by using qualitative methodologies, which often lack evidence in quantitative terms, and she places the qualitative researchers “somewhere between fact and conjecture” (p. 15). Thorne says that interpretive description was a way for researchers to liberate themselves from using strictly normative methodologies or as Sandelowski (2000) stated, a way to be free from the “tyranny of method” (p. 334) in favour of a less directed method. Interpretive description (Thorne, Reimer Kirkham, & MacDonald-Eames, 1997; Thorne, Reimer Kirkham, & O'Flynn-Magee, 2004) is a qualitative investigation of a clinical phenomenon of interest to the discipline. There are various disciplines in a clinic that will also direct the purpose of research questions derived from the discipline. Themes and patterns are captured within subjective perceptions, and they generate an interpretive description presenting a clinical understanding. Researchers have made efforts to “build methods that are grounded in our own epistemological foundations, adhere to the systematic reasoning of our own discipline and yield legitimate knowledge for our practice” (Thorne et al., 1997, p. 172). More recently, interpretive description has been presented as a methodology for all the applied disciplines (Thorne, 2008), aimed at generating knowledge relevant for the clinical context of applied health disciplines.

Looking at interpretive description (Thorne, 2008) some thoughts come to mind. What is the benefit of this “new” method? What is new? Interpretive description draws on methodological principles developed for the theoretical purposes of various social science traditions (Glaser & Strauss, 1967; Lincoln & Guba, 1985; Miles & Huberman, 1994).

Interpretive description is strongly influenced by and borrows several aspects from grounded theory, naturalistic inquiry, ethnography, and phenomenology, when presenting designs, samples, data collection, and analysis. The influence from grounded theory is made clear by using questions such as; “What is happening here?” and “What am I learning about this?” These are questions that are intended to stimulate more coherent analytic frameworks for interpretive description.

What is the purpose of creating a method that is very similar to grounded theory? If the answer is that the researchers prefer to comprehend the data as individuals rather than as concepts, then modification of grounded theory by Charmaz (2006/2008), focusing on people, would be a suitable choice. Perhaps, it is the perspective of phenomenology that is the issue. Interpretive descriptions depend on the subjective experiences of individuals while learning from broader patterns within the phenomenon under study (Thorne, 2008). Why not use interpretive phenomenology? Even qualitative content analysis could be used, bringing forth both interpretation and description.

Thorne means that there is a matter of quality, that there is a link between research and clinical practice. Is the issue perhaps to apply methodological principles to practice and then be able to present valid findings to clinicians? How can interpretive description meet this requirement? The other methods mentioned above (grounded theory, content analysis, phenomenology) are morally defensible, are used in clinical research, and clinicians understand the findings and are able to understand and use the knowledge presented in their own reality.

Is it due to the morally defensible strengths of the other methods that various verification strategies are borrowed and used in interpretive descriptions, such as concurrent data collection and analysis, constant comparative analysis, and iterative analysis? These strategies are used to locate the findings within the framework of the existing body of knowledge.

Validity has to be considered, especially the validity of findings in an interpretive description study, and this is done by reflecting upon the extent to which they match or enlarge the clinical hunches of clinicians familiar with the phenomenon under study. That is why researchers performing interpretive description throughout the analytic process share emerging findings with clinicians (Maheu & Thorne, 2008, p. 557). This is also a well-known verification strategy.

Researchers using interpretive description also move beyond description to develop “coherent conceptual description that taps thematic patterns and commonalities believed to characterise the phenomenon that is being studied. Interpretive description also accounts for the inevitable individual variations within them … understandings of clinical phenomena that illuminate their characteristics, patterns and structure in some theoretically useful manner” (Thorne et al., 2004, p. 3).This seems quite similar to linking concepts and building theory and causal interpretive explanations.

Thorne says that interpretive description was created to free researchers from methodological orthodoxy. The basic guidelines presented direct you on what to do, starting with a critical analysis of existing theoretical and clinical knowledge within the discipline. This gives the starting conceptual framework. No detailed procedures are presented, instead there are general criteria to be used by the researcher to examine design decisions and adaptations of qualitative methods. Thorne (2008) presents a definition of interpretive description:

Interpretive description is an approach to knowledge generation that straddles the chasm between objective neutrality and the abject theorizing extending form of understanding that is of partial importance to the applied discipline within the context of their distinctive social mandates. It responds to the imperative for informed action within the admittedly imperfect scientific foundation that is the lot of human science. (p. 26)

Looking at interpretive description, reading texts written about the method, and reading articles using interpretive description in performed studies, it appears that grounded theory is to be interpretive description's epistemological foundation. So is this a new method (a qualitative research approach)? My answer is NO. I will go out on a limb and say, interpretive description seems in many ways to be part of the movement of grounded theory.

*Carina Berterö* Chief Editor Linköping University Linköping, Sweden
